# Ultra-late response (> 24 weeks) to anti-CGRP monoclonal antibodies in migraine: a multicenter, prospective, observational study

**DOI:** 10.1007/s00415-023-12103-4

**Published:** 2024-01-17

**Authors:** Piero Barbanti, Cinzia Aurilia, Gabriella Egeo, Stefania Proietti, Florindo D’Onofrio, Paola Torelli, Marco Aguggia, Davide Bertuzzo, Cinzia Finocchi, Michele Trimboli, Sabina Cevoli, Giulia Fiorentini, Bianca Orlando, Maurizio Zucco, Laura Di Clemente, Ilaria Cetta, Bruno Colombo, Monica Laura Bandettini di Poggio, Valentina Favoni, Licia Grazzi, Antonio Salerno, Antonio Carnevale, Micaela Robotti, Fabio Frediani, Claudia Altamura, Massimo Filippi, Fabrizio Vernieri, Stefano Bonassi, ERT; for the Italian Migraine Registry study group

**Affiliations:** 1 Headache and Pain Unit, IRCCS San Raffaele Roma, Via Della Pisana 235, 00163 Rome, Italy; 2 San Raffaele University, Rome, Italy; 3 Clinical and Molecular Epidemiology, IRCCS San Raffaele Roma, Rome, Italy; 4 Headache Center Neurology Unit, San Giuseppe Moscati Hospital, Avellino, Italy; 5 Unit of Neurology, Department of Medicine and Surgery, Headache Center, University of Parma, Parma, Italy; 6 Headache Center Cardinal Massaia, Asti, Italy; 7 Divisione di Neurologia, Ospedale San Paolo, ASL 2 Savonese, Savona, Italy; 8 University Magna Graecia, Catanzaro, Italy; 9 IRCCS Istituto delle Scienze Neurologiche di Bologna, Bologna, Italy; 10 Headache Center, San Camillo-Forlanini Hospital, Rome, Italy; 11 Neurology Unit, IRCCS San Raffaele Scientific Institute, Vita-Salute San Raffaele University, Milan, Italy; 12 IRCCS Ospedale Policlinico San Martino, Genoa, Italy; 13 Neuroalgology Unit, Headache Center Fondazione, IRCCS Istituto Neurologico“Carlo Besta”, Milan, Italy; 14 Headache Center San Giovanni Addolorata Hospital, Rome, Italy; 15 Headache Center San Filippo Neri Hospital, Rome, Italy; 16 Headache Center, ASST Santi Paolo Carlo, Milan, Italy; 17 Headache and Neurosonology Unit, Policlinico Universitario Campus Bio-Medico, Rome, Italy

**Keywords:** Migraine, Treatment, Anti-CGRP mAbs, Responder, Late response, Ultra-late response, Real-life

## Abstract

**Objective:**

Nearly 60% of migraine patients treated with monoclonal antibodies (mAbs) targeting the calcitonin gene-related peptide (CGRP) pathway experience a ≥ 50% reduction in monthly migraine days (MMD) at 12 weeks compared to baseline *(responders)*. However, approximately half of the patients not responding to anti-CGRP mAbs ≤ 12 weeks do respond ≤ 24 weeks (*late responders)*. We assessed frequency and characteristics of patients responding to anti-CGRP mAbs only > 24 weeks (*ultra-late responders*).

**Methods:**

In this multicenter (*n* = 16), prospective, observational, real-life study, we enrolled all consecutive adults affected by high-frequency episodic migraine (HFEM: ≥ 8 days/month) or chronic migraine (CM), with ≥ 3 prior therapeutic failures, treated with any anti-CGRP mAbs for ≥ 48 weeks. We defined *responders* patients with a ≥ 50% response rate ≤ 12 weeks, *late responders* those with a ≥ 50% response rate ≤ 24 weeks, and *ultra-late responders* those achieving a ≥ 50% response only > 24 weeks.

**Results:**

A total of 572 migraine patients completed ≥ 48 weeks of anti-CGRP mAbs treatment. *Responders* accounted for 60.5% (346/572), *late responders* for 15% (86/572), and *ultra-late responders* for 15.7% (90/572). Among *ultra-late responders*, 7.3% (42/572) maintained the ≥ 50% response rate across all subsequent time intervals (weeks 28, 32, 36, 40, 44, and 48) and were considered *persistent ultra-late responders*, while 8.4% (48/572) missed the ≥ 50% response rate at ≥ 1 subsequent time interval and were classified as *fluctuating ultra-late responders*. Fifty patients (8.7%) did not respond at any time interval ≤ 48 weeks. *Ultra-late responders* differed from *responders* for higher BMI (*p* = 0.033), longer duration of medication overuse (*p* < 0.001), lower NRS (*p* = 0.017) and HIT-6 scores (*p* = 0.002), higher frequency of dopaminergic symptoms (*p* = 0.002), less common unilateral pain—either alone (*p* = 0.010) or in combination with UAS (*p* = 0.023), allodynia (*p* = 0.043), or UAS and allodynia (*p* = 0.012)—a higher number of comorbidities (*p* = 0.012), psychiatric comorbidities (*p* = 0.010) and a higher proportion of patients with ≥ 1 comorbidity (*p* = 0.020).

**Conclusion:**

Two-thirds of patients not responding to anti-CGRP mAbs ≤ 24 weeks do respond later, while *non-responders* ≤ 48 weeks are quite rare (8.7%). These findings suggest to rethink the duration of migraine prophylaxis and the definition of resistant and refractory migraine, currently based on the response after 2–3 months of treatment.

**Supplementary Information:**

The online version contains supplementary material available at 10.1007/s00415-023-12103-4.

## Introduction

Migraine is a chronic evolutive neurological disorder that is often under-estimated and under-treated [1, 2]. Early and appropriate treatment for migraine is recommended not only to improve patients’ quality of life but also to prevent disease progression and to reduce the risk of medication overuse. Nonetheless, despite more than one-fourth of migraine patients being eligible for prophylaxis [3, 4], only a small proportion of them use preventive medications (2%-12% in Europe, 16.8% in US). Reasons for this include low disease awareness, barriers to migraine diagnosis and treatment, and the suboptimal efficacy and low tolerability of conventional drugs (standard of care (SoC): beta-blockers, anticonvulsants, tricyclics, calcium-channel antagonist) which limit their use to relatively short periods (4–6 months) [5].

The availability of monoclonal antibodies targeting the CGRP pathway (anti-CGRP mAbs)—medications characterized by an unprecedented balance between efficacy and tolerability—has prompted to reconsider the paradigm of migraine prevention, recommending a much longer prophylactic treatment period (12–18 months) [6]. Notably, studies on extended treatments with anti-CGRP mAbs has brought into question the validity of evaluating efficacy at the conventional 3-month interval, typically employed in randomized controlled trials (RCTs). This threshold might be misleading since a considerable proportion (55%) of individuals not responding to anti-CGRP at 12 weeks do achieve a ≥ 50% response within 24 weeks and implies that 1 out of 5 migraine patients is indeed a *late responder* [7]. Late response to migraine preventative medication has pathophysiological and clinical implications, suggesting that in a large proportion of patients central desensitization may require a longer time, and emphasizing the need for extended treatment duration in these individuals.

The reversal of mechanisms involved in migraine progression and chronicization might be even slower in some patients. To explore this hypothesis, we conducted a multicenter, prospective, real-life study aimed at assessing the presence of *ultra-late responders* (> 24 weeks) to anti-CGRP mAbs in a large cohort of patients affected by high-frequency episodic migraine (HFEM: 8–14 days/month) or chronic migraine (CM) with multiple prior therapeutic failures.

## Methods

This is an ongoing multicenter, cohort, real-life study involving 16 headache centers across 7 Italian regions. It started on December 2018 as part of the I-NEED (Italian NEw migrainE Drugs database) project, which is under the umbrella of the Italian Migraine Registry (I-GRAINE).

We enrolled all consecutive adult patients affected by HFEM or CM, with ≥ 3 prior therapeutic failures and a MIDAS score > 11—according to the rules of the Italian Medicines Agency—treated with erenumab (70 mg or 140 mg every 28 days), galcanezumab (120 mg following a loading dose of 240 mg every 30 days), or subcutaneous fremanezumab (225 mg every 30 days or 675 mg every 90 days) for ≥ 48 weeks [8]. AntiCGRP mAbs prescription was made based on drug market availability, physician’s choice, or patient’s preference. We excluded patients with fewer than eight migraine days per month, use of onabotulinum toxin A during the previous 12 weeks, prior use of anti-CGRP mAbs or significant cardiovascular or cerebrovascular disorders. No additional preventive medications were started during the observation period.

The study received approval from the IRCCS San Raffaele Roma Institutional Review Board (RP 11/2018) and was mutually recognized by the other local Institutional Review Boards. The study was not preregistered on any study registry site.

After providing written informed consent, all patients were interviewed face-to-face by specifically trained, board-certified headache specialists using a semi-structured, web-based questionnaire that carefully explored sociodemographic and clinical characteristics [9]. In addition to common migraine features (e.g., frequency, pain severity, disability), we also assessed the presence of allodynia, unilateral cranial autonomic symptoms (UAS, defined as ≥ 1 of the following unilateral symptoms during the migraine attack: lacrimation, eye redness, nasal congestion, ptosis, eyelid swelling, miosis or forehead/facial sweating) [10], and presence of dopaminergic symptoms (defined as ≥ 1 of the following symptoms during prodromes, headache stage or postdromes: yawning, somnolence, nausea, vomiting, mood changes, fatigue or diuresis) [11].

We defined *responders* patients with a ≥ 50% reduction from baseline in monthly migraine days (MMDs) in HFEM or monthly headache days (MHDs) in CM at weeks 9–12, *late responders* those achieving a ≥ 50% reduction between weeks 13–16 and 21–24, and *ultra-late responders* those individuals reaching a ≥ 50% reduction only > 24 weeks. The term “headache day” refers to any headache day, including both migraine-like and tension-type like days. We named *persistent* ultra-late responders those patients who remained ≥ 50% responders at all subsequent time intervals (i.e., weeks 28, 32, 36, 40, 44, and 48) and *fluctuating* ultra-late responders those not maintaining the 50% response rate at ≥ 1 subsequent time interval. Patients were asked to detail MMDs or MHDs during a 28-day run-in period and across the study using a paper–pencil headache diary.

The primary aim of the study was to assess the presence of *ultra-late responders* to anti-CGRP mAbs. Additional objectives of the study included profiling *ultra-late responders* and investigating differences with *responders*.

## Statistical analysis

Convenience sampling was used since previous studies from the same registry demonstrated that the number of patients included in our study was large enough to allows a reliable estimation of response and to compare different subclasses or response. The p-values should be considered as an index of the credibility rather than a test of hypothesis. Categorical variables were represented as numbers and percentages, and their analysis was conducted using the χ2 test or Fisher's exact test when appropriate. Quantitative variables that followed a normal distribution, they were presented as means and standard deviations, otherwise median and interquartile range (IQR) were shown, and nonparametric tests were applied.

To assess changes from baseline in patients at different time points, a paired t-test was utilized. When comparing groups with only one factor and two categories, an independent t-test was applied. If there were more than two categories, ANOVA was used. The proportion of missing data was < 10% for all variables except psychiatric comorbidities and BMI (12% and 11%, respectively), and therefore, a complete case analysis was performed. The study was exploratory in nature, aiming to explore a wide range of potential associations and hypotheses, rather than testing specific pre-defined hypotheses. Therefore, we decided not to apply Bonferroni correction, which is more suitable for confirmatory studies with a limited number of a priori hypotheses, while in exploratory research an overly stringent correction like Bonferroni might stifle the identification of potentially important findings. Sensitivity analyses were carried out excluding one clinical center at a time and examining the impact of the removal on the summary treatment effect. A p-value of less than 0.05 was considered statistically significant. All statistical analyses were performed using SPSS 28.0 software.

## Results

**Fig. 1:**
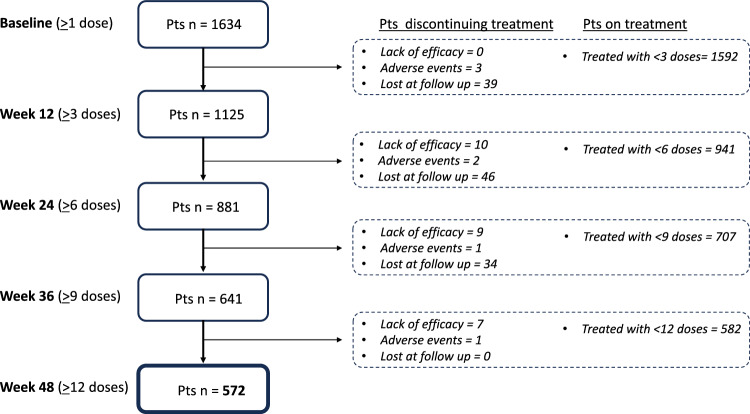
Flow chart of the study population

As of March 31, 2023, 1634 patients had received at least 1 dose of anti-CGRP mAbs, while 572 had completed ≥ 48 weeks of treatment, having received at least 12 doses (HFEM/CM:154/418; F/M:432/140; mean age: 48.2 years; erenumab/fremanezumab/galcanezumab: 527/40/5). No patient switched from one anti-CGRP mAb to another, as this is not permitted under current Italian reimbursement rules. Figure 1 depicts the progression of patients along the study. Patients with CM differed from those affected by HFEM in terms of higher body mass index (BMI), monthly analgesic medication use, HIT-6 and MIDAS scores, and a higher frequency of UAS, and treatment failures (Table 1).

**Table 1:** Demographic and clinical features of patients with high-frequency episodic migraine (HFEM) or chronic migraine (CM)

	Number (%) or mean ± SD			
	All patients	HFEM	CM	*p*-value
Patients	572	154	418	–
Age, yrs	48.2 ± 10.6	49.0 ± 10.6	47.9 ± 10.7	0.252
Females	432 (75.5)	116 (75.3)	316 (75.6)	1.000
BMI	23.2 ± 3.6	22.6 ± 2.7	23.4 ± 3.8	0.022
Age onset	17.7 ± 8.8	18.3 ± 10.2	17.5 ± 8.3	0.313
MMDs at baseline	–	10.3 ± 2.3		–
MHDs at baseline	–		23.4 ± 5.6	–
Monthly analgesic medications	23.9 ± 20.1	12.9 ± 6.4	28.0 ± 21.9	< 0.001
Medication overuse	370 (64.7)	–	370 (88.5)	–
Medication overuse duration, months	49.5 ± 90.8	–	49.5 ± 90.8	–
NRS score	7.6 ± 1.3	7.4 ± 1.5	7.6 ± 1.3	0.075
Unilateral pain	325 (56.9)	95 (62.1)	230 (55.0)	0.157
UAS	301 (52.6)	68 (44.2)	233 (44.7)	0.018
Allodynia	331 (57.9)	81 (52.6)	250 (59.8)	0.146
Dopaminergic symptoms	379 (66.3)	107 (69.5)	272 (65.1)	0.374
Unilateral pain + UAS	185 (32.3)	46 (29.9)	139 (33.2)	0.471
Unilateral pain + allodynia	191 (33.5)	51 (33.1)	140 (33.5)	0.971
Unilateral pain + UAS + allodynia	135 (23.6)	31 (20.1)	104 (24.9)	0.250
HIT-6 score	65.7 ± 10.0	64.1 ± 10.9	66.3 ± 9.6	0.026
MIDAS	81.6 ± 56.6	57.6 ± 56.2	91.7 ± 54.3	0.030
Triptan responders	366 (75.6)	106 (80.3)	260 (73.9)	0.177
Acute treatment NSAIDs Triptans Analgesic combinations Opioids	567 (99.1) 287 (50.6) 439 (77.4) 95 (16.8) 9 (1.6)	154 (100) 75 (48.7) 117 (76.0) 19 (12.3) –	413 (98.8) 212 (51.3) 322 (78.0) 76 (18.4) 9 (2.2)	0.909 0.396
Prior treatment failures *1–2* *3–4* > *5*	4.9 ± 1.9 42 (7.4) 183 (32.4) 340 (60.2)	4.3 ± 1.7 21 (13.8) 62 (40.8) 69 (45.4)	5.2 ± 1.9 21 (5.1) 121 (29.3) 271 (65.6)	< 0.001 < 0.001
Pts using concomitant prophylaxis	298 (52.1)	80 (51.9)	218 (52.2)	1.000
Comorbidities	1.0 ± 1.1	1.1 ± 1.2	1.0 ± 1.1	0.245
Pts with ≥ 1 comorbidity	347 (60.7)	97 (63.0)	250 (59.8)	0.553
Pts with psychiatric comorbidities	185 (32.3)	48 (31.2)	137 (32.8)	0.792
Onabotulinum toxin A responders*	22 (8.4)	7 (13.5)	15 (7.2)	0.195
Erenumab	527 (92.1)	138 (89.6)	389 (93.1)	–
Galcanezumab	5 (0.8)	2 (1.3)	3 (0.7)	–
Fremanezumab	40 (7.0)	14 (9.1)	26 (6.2)	–

*BMI* body mass index; *MMD* monthly migraine day; *MHD* monthly headache day; *NRS* numerical rating scale; *UAS* unilateral cranial autonomic symptoms; HIT-6 headache impact test-6; *MIDAS* migraine disability assessment scale; *NSAIDs* non-steroidal anti-inflammatory drugs

**Fig. 2:**
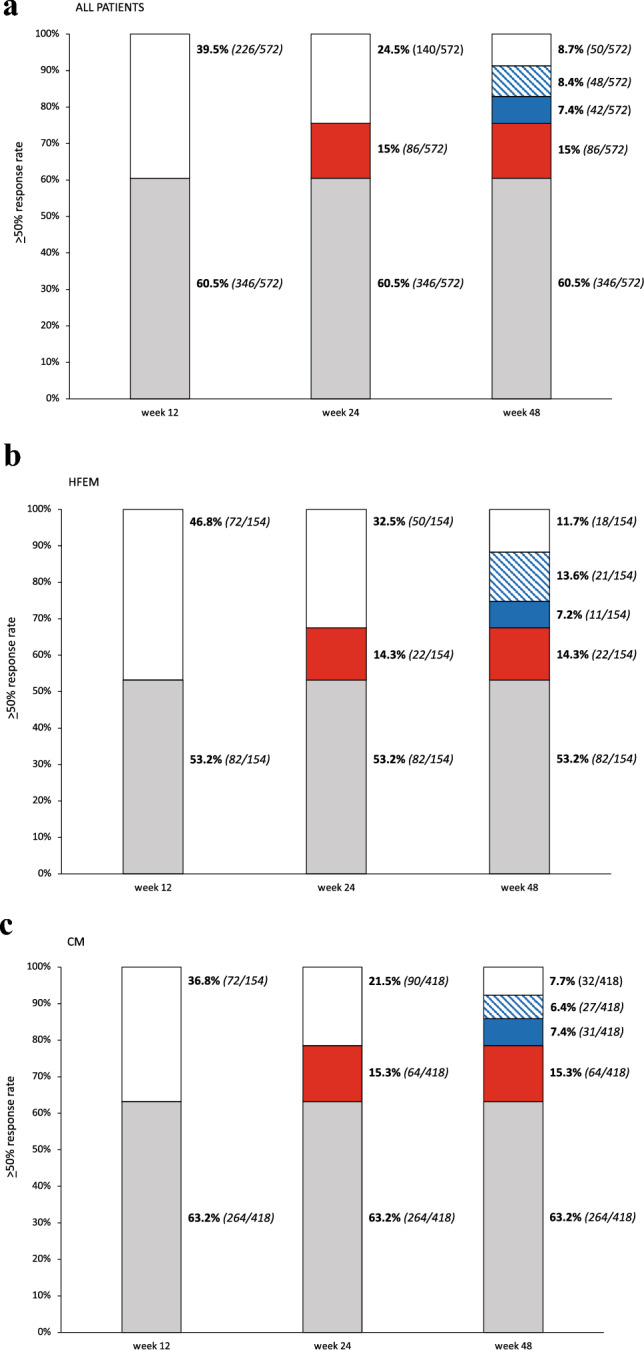
Proportion of patients with a 50% or greater reduction in monthly migraine/headache days following treatment with antiCGRPmAbs in all patients (**a**), high-frequency episodic migraine (HFEM) (**b**) and chronic migraine (CM) (**c**) Light gray bars indicate *responders* (≤ 12 weeks), white bars *non-responders*, red bars *late-responders* (> 12 weeks), blue bars *persistent ultra-late responders* (> 24 weeks; stable ≥ 50% response across following time intervals), and dashed blue bars *fluctuating ultra-late responders* (> 24 weeks; instable ≥ 50% response across following time intervals)

**Fig. 3:**
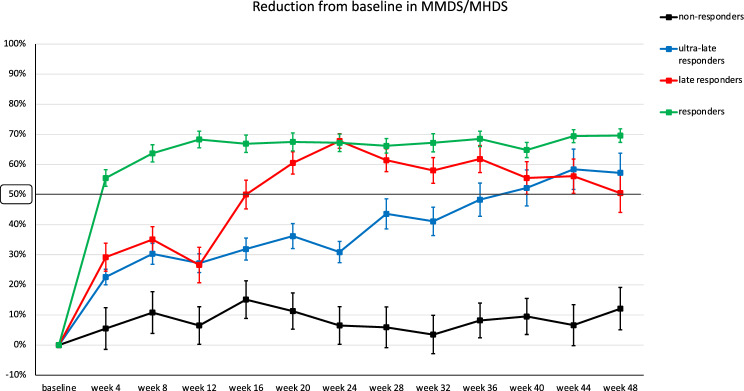
Mean (SE) change from baseline in monthly migraine days (MMDs)/monthly headache days (MHDs) in responders (< 12 weeks), late-responders (> 12 weeks), ultra-late responders (> 24 weeks) and non-responders during 0–48 treatment weeks. SE: standard error

Out of the 572 patients who completed ≥ 48 weeks of treatment, 346 (60.5%) showed a ≥ 50% response rate ≤ 12 weeks (*responders*), while 86 (15%) who did not respond ≤ 12 weeks achieved a ≥ 50% response within 24 weeks (*late-responders*). Ninety of the 140 subjects non-responding ≤ 24 weeks (15.7%), became treatment responder within 48 weeks (*ultra-late responders*) (Table 2). Forty-two *ultra-late responders* (7.3%) maintained the ≥ 50% response rate across all subsequent time intervals (i.e., weeks 28, 32, 36, 40, 44, and 48) and were considered *persistent ultra-late responders*. Conversely*,* 48 ultra-late responders (8.4%) missed the ≥ 50% response rate at ≥ 1 subsequent time interval and were classified as *fluctuating ultra-late responders*. Fifty patients (8.7%) did not respond at any time interval ≤ 48 weeks (Fig. 2). Figure 3 illustrates the reduction from baseline in MMDs/MHDs across 48 treatment weeks in *responders*, *late responders*, *ultra-late responders,* and *non-responders*. Adverse events, calculated for subject who had received at least one dose of mAbs, occurred in 3.9% (63/1634) of the patients. The most common adverse events were constipation (2.4%), injection site erythema (0.6%), and back pain (0.6%). Serious adverse events were reported by 3 patients (0.18%) who were affected by CM with medication overuse and treated with erenumab. Two of these individuals manifested non-ST segment elevation myocardial infarction, which was considered unrelated to the treatment, while one patient developed a treatment-related paralytic ileus 20 days after receiving the first erenumab 70 mg dose. Only the three patients with serious adverse events discontinued the treatment.

**Table 2:** Demographic and clinical features of responders (R), late responders (LR), ultra-late responders (ULR), and non-responders (NR)

	Number (%) or mean ± SD				
	R	LR	ULR	NR	*p*-value
Patients	346 (60.5)	86 (15)	90 (15.7)	50 (8.7)	-
Females	268 (77.5)	63 (73.3)	69 (76.7)	32 (64.0)	0.204
Age, yrs	48.3 ± 10.4	46.8 ± 11.5	48.6 ± 10.1	48.4 ± 10.9	0.612
BMI	23.0 ± 3.1	23.1 ± 4.4	23.8 ± 4.2	23.6 ± 3.2	0.188
Age at onset	17.9 ± 8.8	15.6 ± 7.0	18.5 ± 10.2	18.0 ± 9.1	0.121
HFEM CM	82 (23.7) 264 (76.3)	22 (25.6) 64 (74.4)	32 (35.6) 58 (64.4)	18 (36.0) 32 (64.0)	0.060
MMDs at baseline	18.0 ± 7.1	17.1 ± 7.0	16.4 ± 7.4	17.5 ± 8.3	0.318
MHDs at baseline	20.5 ± 7.1	19.2 ± 7.4	19.7 ± 8.1	19.1 ± 8.1	0.336
Monthly analgesic medications	23.0 ± 20.3	23.5 ± 17.5	25.6 ± 20.3	27.8 ± 22.8	0.362
Medication overuse	228 (86.4)	58 (90.6)	52 (89.7)	32 (100.0)	0.127
Medication overuse duration, months	35.9 ± 81.7	62.5 ± 87.7	78.4 ± 110.7	82.5 ± 107.4	< 0.001
NRS score	7.7 ± 1.3	7.7 ± 1.4	7.3 ± 1.3	7.3 ± 1.5	0.052
Unilateral pain	215 (62.3)	46 (53.5)	42 (46.7)	22 (44.0)	0.008
UAS	190 (54.9)	43 (50.0)	46 (51.1)	22 (44.0)	0.468
Allodynia	206 (59.5)	48 (55.8)	50 (55.6)	27 (54.0)	0.789
Unilateral pain + UAS	127 (36.7)	27 (31.4)	21 (23.3)	10 (20.0)	0.019
Unilateral pain + allodynia	130 (37.6)	24 (27.9)	23 (25.6)	14 (28.0)	0.067
Unilateral pain + UAS + allodynia	97 (28.0)	16 (18.6)	13 (14.4)	9 (18.0)	0.017
Dopaminergic symptoms	212 (61.3)	57 (66.3)	71 (78.9)	39 (78.0)	0.004
HIT-6 score	66.9 ± 7.7	64.2 ± 13.9	63.7 ± 11.6	64.2 ± 11.9	0.009
MIDAS score	80.6 ± 62.8	66.5 ± 20.0	92.5 ± 38.5	99.5 ± 44.3	0.795
Triptan responders	229 (76.8)	52 (60.5)	55 (78.6)	30 (71.4)	0.547
Prior treatment failures 1–2 3–4 > 5	4.8 ± 1.9 30 (8.7) 124 (36.0) 190 (55.2)	5.4 ± 2.6 6 (7.1) 23 (27.4) 55 (65.5)	5.0 ± 1.8 5 (5.7) 24 (27.3) 59 (67.0)	5.4 ± 1.9 1 (2.0) 12 (24.5) 36 (73.5)	0.130 0. 098
Pts using concomitant prophylaxis	178 (51.4)	40 (46.5)	54 (60.0)	26 (52.0)	0.336
Comorbidities	0.9 ± 1.1	1.0 ± 1.1	1.3 ± 1.2	1.3 ± 1.2	0.038
Pts with ≥ 1 comorbidity	197 (56.9)	52 (60.5)	64 (71.1)	34 (68.0)	0.064
Pts with psychiatric comorbidities	104 (30.1)	24 (27.9)	40 (44.4)	17 (34.0)	0.053
Onabotulinum toxin A responders*	11 (8.0)	2 (4.3)	5 (10.4)	4 (13.8)	0.811
Erenumab	311 (89.9)	82 (95.3)	87 (96.7)	48 (96.0)	–
Galcanezumab	4 (1.2)	–	–	–	–
Fremanezumab	31 (9.0)	4 (4.7)	3 (3.3)	2 (4.0)	–

*HFEM* high frequency episodic migraine; *CM* chronic migraine; *R* responders; *ULR* ultra-late responders; *BMI* body mass index; *MMD* monthly migraine day; *MHD*, monthly headache day; *UAS* unilateral cranial autonomic symptoms; *NRS* numerical rating scale; *HIT-6* Headache Impact Test-6; *MIDAS* migraine disability assessment scale. ^*^Proportion calculated on the 261 subjects who were treated with onabotulinum toxin A

A comprehensive analysis comparing *responders*, *late-responders*, *ultra-late responders*, and *non-responder*s for several clinical and demographic parameters, showed significant differences in medication overuse duration, unilateral pain (either alone or associated with UAS, or UAS and allodynia), dopaminergic symptoms, and HIT-6 score and number of comorbidities (Table 2).

*Ultra-late responders* differed from *responders* in terms of higher BMI (+ 0.86, 95% CI [0.07;1.66]; *p* = 0.033), longer medication overuse duration (median 24 vs 6, IQR 3–123 vs 1–15; *p* < 0.001), lower NRS (− 0.36, 95% CI [− 0.66.0; − 0.06]; *p* = 0.017), and HIT-6 scores (− 3.16, 95% CI [− 5.16; − 1.15]; *p* = 0.002). They also exhibited a higher frequency of dopaminergic symptoms (+ 17.6%, 95% CI [0.07;0.27]; *p* = 0.002), less common unilateral pain either alone − 15.7%, 95% CI [− 26.9; − 4.1]; *p* = 0.010) or in combination with UAS (− 13.5%, 95% CI [− 23.1; − 2.9]; *p* = 0.023), allodynia (− 12.1%, 95% CI [− 22.0; − 1.3]; *p* = 0.043), or UAS and allodynia (− 13.7%, 95% CI [− 21.8;-4.3]; *p* = 0.012), a higher number of comorbidities (+ 0.32, 95% CI [0.07;0.57]; *p* = 0.012), psychiatric comorbidities (+ 14.4%, 95%CI [3.1;25.6]; p = 0.010) and a higher proportion of patients with at least 1 comorbidity (+ 14.2%, 95% CI [3.1;24.4]; *p* = 0.020) (Table 3). Differences between *ultra-late responders* and *responders* were primarily driven by CM patients, as detailed in the supplemental Table 1.

**Table 3:** Comparison of demographic and clinical features of responders (R) and ultra-late responders (ULR)

	All patients (*n* = 572)				
	*R*	ULR	*p*-value	Difference	95% CI
Patients	346 (79.4)	90 (20.6)	-		
Females	268 (77.5)	69 (76.7)	0.873		
Age, yrs	48.3 ± 10.4	48.6 ± 10.1	0.839		
BMI	23.0 ± 3.1	23.8 ± 4.2	0.033	+ 0.86	[0.07;-1.66]
Age at onset	18.0 ± 8.8	18.5 ± 10.1	0.614		
MMDs at baseline	18.0 ± 7.1	16.4 ± 7.4	0.066		
MHDs at baseline	20.5 ± 7.1	19.7 ± 8.1	0.344		
Analgesics per month	23.0 ± 20.3	25.6 ± 20.3	0.293		
Medication overuse	228 (86.3)	52 (89.7)	0.500		
Medication overuse duration, months^#^	6 [1–15]	24 [3–123]	< 0.001 [Z = -3.805]		
NRS score at baseline	7.7 ± 1.3	7.3 ± 1.3	0.017	– 0.36	[– 0.66;– 0.06]
Unilateral pain	215 (62.3)	42 (46.7)	0.010	– 15.7%	[– 26.9;– 4.10]
UAS	190 (54.9)	46 (51.1)	0.519		
Allodynia	206 (59.5)	50 (55.6)	0.494		
Unilateral pain + UAS	127 (36.8)	21 (23.3)	0.023	– 13.5%	[– 23.1;– 2.90]
Unilateral pain + allodynia	130 (37.7)	23 (25.6)	0.043	– 12.1%	[– 22.0;– 1.30]
Unilateral pain + UAS + allodynia	97 (28.1)	13 (14.4)	0.012	– 13.7%	[– 21.8;– 4.30]
Dopaminergic symptoms	212 (61.3)	71 (78.9)	0.002	+ 17.6%	[7.20;26.9]
HIT-6 score at baseline	66.9 ± 7.7	63.7 ± 11.6	0.002	– 3.16	[– 5.16;– 1.15]
MIDAS score at baseline^#^	70 [29.5–114]	99 [69.2–120]	0.364		
Triptan responders	229 (76.8)	55 (78.5)	0.880		
Prior treatment failures *1–2* *3–4* > *5*	4.8 ± 1.9 30 (8.7) 124 (36.1) 190 (55.2)	5.0 ± 1.8 5 (5.7) 24 (27.3) 59 (67.0)	0.421 0.131		
Pts using concomitant prophylaxis	178 (51.4)	54 (60.0)	0.183		
Comorbidities	0.9 ± 1.1	1.3 ± 1.2	0.012	+ 0.32	[0.07;0.57]
Pts with ≥ 1 comorbidity	197 (56.9)	64 (71.1)	0.020	+ 14.2%	[3.10;24.4]
Pts with psychiatric comorbidities	104 (30.1)	40 (44.4)	0.010	+ 14.4%	[3.10;25.6]
Onabotulinum toxin A responders*	11 (8.0)	5 (10.4)	0.796		
Erenumab	311 (89.9)	87 (96.7)	–		
Galcanezumab	4 (1.2)	–	–		
Fremanezumab	31 (9.0)	3 (3.3)	–		

*BMI* body mass index; *MMD* monthly migraine day; *MHD* monthly headache day; *UAS* Unilateral cranial autonomic symptoms; *NRS* Numerical Rating Scale; *HIT-6* headache impact test-6; *MIDAS* migraine disability assessment scale

^#^median [interquartile range, IQR]; ^*^Proportion calculated on the 261 subjects who were treated with Onabotulinum toxin A

## Discussion

The present multicenter, prospective, real-life study documents that 15.7% of patients affected by HFEM or CM with multiple prior therapeutic failures are *ultra-late responders* to anti-CGRP mAbs, achieving a *persistent* or *fluctuating* ≥ 50% response rate only after 24 weeks of treatment. This indicates that two-thirds of migraine patients who do not respond at 24 weeks do respond later.

U*ltra-late responders* do indeed show an early, gradual, progressive reduction in MMDs/MHDs which reaches a ≥ 30% response rate—a clinically meaningful endpoint, at least in CM—between weeks 8 and 24 [12] (Fig. 3). Conversely, *non-responders* remain persistently below a ≥ 20% response rate throughout the entire 48-week treatment period. Pooling together *responders*, *late responders*, and *ultra-late responders*, the proportion of patients with a ≥ 50% response to anti-CGRP mAbs after 1 year of treatment is 91.3%, while the occurrence of *non-responders* is quite rare (8.7%).

Why do some migraine patients affected by HFEM or CM respond very late to anti-CGRP mAbs remains unclear. Anti-CGRP mAbs act centripetally, desensitizing trigeminal peripheral nociceptors and subsequently reversing central sensitization. This mechanism becomes clinically evident within 12 weeks, on average, in nearly 60% of patients. However, substantial inter-individual differences may occur. For instance, patients experiencing intense trigemino-vascular activation symptoms (such as unilateral pain, either alone or in association with UAS) appear to be particularly sensitive to anti-CGRP mAbs, exhibiting a notably rapid and high response rate, probably linked to the heightened sensitization of trigeminal nociceptors [9, 13].

By analyzing the migraine phenotype and clinical history of *ultra-late responders*, we hypothesize that the pathophysiological mechanisms underlying the delayed therapeutic effects of anti-CGRP mAbs could involve reduced trigeminal sensitization, increased central CGRP activity, or both. Reduced sensory activation in *ultra-late responders*—inferred from their milder and less frequently unilateral pain—could slow down the therapeutic effect of anti-CGRP mAbs. Milder pain in *ultra-late responders* could also account for the lower HIT-6 score, as this questionnaire is highly sensitive to migraine intensity [14].

On the other hand, the complex clinical picture of *ultra-late responders* suggests an increased central CGRP activity as indicated by higher BMI values, more numerous comorbidities, psychiatric disorders, dopaminergic symptoms, and longer duration of medication overuse compared to *responders* [11, 15–17]. Therefore, a longer time may be needed to counteract increased central sensitization, delaying the onset of the therapeutic effects of anti-CGRP mAbs.

*Ultra-late response* to migraine prophylaxis is a relatively unexplored field. Common preventative agents are typically used for short treatment periods (3–6 months) and are often discontinued due to their low tolerability [5]. The introduction of onabotulinum toxin A and anti-CGRP mAbs, medications with a favorable efficacy/tolerability ratio, has paved the way for a new migraine treatment strategy characterized by long-lasting therapies [18, 19]. Prolonged migraine treatment has a solid rationale because migraine progression and chronicization are slowly evolving processes characterized by substantial anatomical, physiological, and biochemical changes in the brain, responsible for increased neuronal excitability [1, 20–22]. Increasing migraine frequency is also known to induce brain adaptive changes spreading from central pain networks to areas controlling non-pain behaviors (neurolimbic pain network), thus explaining the occurrence of common comorbidities (psychiatric disorders, fibromyalgia, irritable bowel disease). Consequently, prolonged migraine prophylaxis is needed to reverse progression mechanisms and consolidate the clinical improvement.

*Late-* and *ultra-late responses* to migraine preventive medications have meaningful implications for the proper clinical management of the disease. Firstly, they prompt to postpone the assessment of the efficacy of migraine prophylaxis, typically fixed at 12 weeks, to (at least) 24 weeks. Secondly, encourage consideration of extending anti-CGRP mAbs treatment beyond 6 months in patients with a ≥ 30% response at week 12. Thirdly, suggests reconsidering the definition of resistance o refractoriness to migraine prevention, which currently consider treatment response at 2 months for SoC, 3 for anti-CGRP mAbs, and 6 for onabotulinum toxin A [23].

Limitations of the study include the fact that we considered only patients affected by HFEM and CM, thus excluding individuals with low attack frequency, and that most of them were treated with erenumab.

Furthermore, longer follow-up studies are required to mitigate the potential bias resulting from the phenomenon of regression to the mean, which could have influenced our results to some extent [24].

The main strengths are the prospective, multicenter design, the large number of patients consecutively enrolled—representative of northern, central, and southern Italy—and their careful clinical characterization through face-to-face interviews with a shared, web-based semi-structured questionnaire.

In conclusion, this study demonstrates the existence of a significant percentage of patients—otherwise considered *non-responders*—who actually respond to treatment with anti-CGRP antibodies only after 24 weeks. 91.3% of migraine patients become *responders* within 48 weeks, with only a small proportion (8.7%) remaining unresponsive. These findings prompt us to rethink the duration of migraine prophylaxis, currently assessed after 2–6 months of treatment, and to reconsider the chronological criteria for defining resistant and refractory migraine. In addition, the presence of late- and ultra-late responders to anti-CGRP mAbs prompts a reconsideration of their reimbursement stopping rules which are presently based on efficacy assessments at 3 or 6 months in various European countries. This time limit appears arbitrary and counterintuitive, as the complexity of mechanisms underpinning migraine progression may necessitate a longer time to observe disease improvement during preventive treatment. The concept of *ultra-late-response* can contribute to a better comprehension of the mechanisms and the time required for desensitizing the migraine brain.

### Supplementary Information

Below is the link to the electronic supplementary material.

Supplementary file1 (DOCX 15 kb)
