# Correction to: Ultra-late response (> 24 weeks) to anti-CGRP monoclonal antibodies in migraine: a multicenter, prospective, observational study

**DOI:** 10.1007/s00415-024-12287-3

**Published:** 2024-04-02

**Authors:** Piero Barbanti, Cinzia Aurilia, Gabriella Egeo, Stefania Proietti, Florindo D’Onofrio, Paola Torelli, Marco Aguggia, Davide Bertuzzo, Cinzia Finocchi, Michele Trimboli, Sabina Cevoli, Giulia Fiorentini, Bianca Orlando, Maurizio Zucco, Laura Di Clemente, Ilaria Cetta, Bruno Colombo, Monica Laura Bandettini di Poggio, Valentina Favoni, Licia Grazzi, Antonio Salerno, Antonio Carnevale, Micaela Robotti, Fabio Frediani, Claudia Altamura, Massimo Filippi, Fabrizio Vernieri, Stefano Bonassi, for the Italian Migraine Registry study group

**Affiliations:** 1 Headache and Pain Unit, IRCCS San Raffaele Roma, Via Della Pisana 235, 00163 Rome, Italy; 2 San Raffaele University, Rome, Italy; 3 Clinical and Molecular Epidemiology, IRCCS San Raffaele Roma, Rome, Italy; 4 Headache Center Neurology Unit, San Giuseppe Moscati Hospital, Avellino, Italy; 5 Unit of Neurology, Department of Medicine and Surgery, Headache Center, University of Parma, Parma, Italy; 6 Headache Center Cardinal Massaia, Asti, Italy; 7 Divisione di Neurologia, Ospedale San Paolo, ASL 2 Savonese, Savona, Italy; 8 University Magna Graecia, Catanzaro, Italy; 9 IRCCS Istituto delle Scienze Neurologiche di Bologna, Bologna, Italy; 10 Headache Center, San Camillo-Forlanini Hospital, Rome, Italy; 11 Neurology Unit, IRCCS San Raffaele Scientific Institute, Vita-Salute San Raffaele University, Milan, Italy; 12 IRCCS Ospedale Policlinico San Martino, Genoa, Italy; 13 Neuroalgology Unit, Headache Center Fondazione, IRCCS Istituto Neurologico“Carlo Besta”, Milan, Italy; 14 Headache Center San Giovanni Addolorata Hospital, Rome, Italy; 15 Headache Center San Filippo Neri Hospital, Rome, Italy; 16 Headache Center, ASST Santi Paolo Carlo, Milan, Italy; 17 Headache and Neurosonology Unit, Policlinico Universitario Campus Bio-Medico, Rome, Italy

**Correction to: Journal of Neurology** 10.1007/s00415-023-12103-4

In the original version of this article, degree “ERT” of author Bonassi Stefano was incorrectly written as author in author group.

Author group which previously read:

Piero Barbanti^1,2^ · Cinzia Aurilia^1^ · Gabriella Egeo^1^ · Stefania Proietti^3^ · Florindo D’Onofrio^4^ · Paola Torelli^5^ · Marco Aguggia^6^ · Davide Bertuzzo^6^ · Cinzia Finocchi^7^ · Michele Trimboli^8^ · Sabina Cevoli^9^ · Giulia Fiorentini^1^ · Bianca Orlando^1^ · Maurizio Zucco^10^ · Laura Di Clemente^10^ · Ilaria Cetta^11^ · Bruno Colombo^11^ · Monica Laura Bandettini di Poggio^12^ · Valentina Favoni^9^ · Licia Grazzi^13^ · Antonio Salerno^14^ · Antonio Carnevale^15^ · Micaela Robotti^16^ · Fabio Frediani^16^ · Claudia Altamura^17^ · Massimo Filippi^11^ · Fabrizio Vernieri^17^ · Stefano Bonassi^2,4^ · ERT; for the Italian Migraine Registry study group.

Should have read:

Piero Barbanti^1,2^ · Cinzia Aurilia^1^ · Gabriella Egeo^1^ · Stefania Proietti^3^ · Florindo D’Onofrio^4^ · Paola Torelli^5^ · Marco Aguggia^6^ · Davide Bertuzzo^6^ · Cinzia Finocchi^7^ · Michele Trimboli^8^ · Sabina Cevoli^9^ · Giulia Fiorentini^1^ · Bianca Orlando^1^ · Maurizio Zucco^10^ · Laura Di Clemente^10^ · Ilaria Cetta^11^ · Bruno Colombo^11^ · Monica Laura Bandettini di Poggio^12^ · Valentina Favoni^9^ · Licia Grazzi^13^ · Antonio Salerno^14^ · Antonio Carnevale^15^ · Micaela Robotti^16^ · Fabio Frediani^16^ · Claudia Altamura^17^ · Massimo Filippi^11^ · Fabrizio Vernieri^17^ · Stefano Bonassi^2,3^ · for the Italian Migraine Registry study group.

And affiliation details for author Stefano Bonassi were incorrectly given as

- ^2^San Raffaele University, Rome, Italy
- ^4^Headache Center Neurology Unit, San Giuseppe Moscati Hospital, Avellino, Italy

but should have been:

- ^2^San Raffaele University, Rome, Italy
- ^3^Clinical and Molecular Epidemiology, IRCCS San Raffaele (not 2 and 4)

