# Supplementary material for: Ultra-late response (> 24 weeks) to anti-CGRP monoclonal antibodies in migraine: a multicenter, prospective, observational study
Source: J Neurol. 2024 Jan 17;271(5):2434–43. doi: 10.1007/s00415-023-12103-4 (PMC11055785; doi:10.1007/s00415-023-12103-4)
Supplement: Supplementary file 1 — Supplementary file1 (DOCX 15 kb) [file 415_2023_12103_MOESM1_ESM.docx]

Supplementary table: Demographic and clinical features of responders (R) and ultra-late responders (ULR) among patients affected by HFEM (high-frequency episodic migraine) and CM (chronic migraine).

|  |  |  |  |  |  |  |  |  |  |
| --- | --- | --- | --- | --- | --- | --- | --- | --- | --- |
|  | HFEM (n=114) | | | CM (n=322) | | |  |  |  |
|  | **R** | **ULR** | ***p-*value** | **R** | **ULR** | ***p-*value** | ***Difference*** | ***95%CI*** |  |
| Patients | 82 (71.9) | 32 (28.1) | - | 264 (82.0) | 58 (18.0) | - |  |  |  |
| Females | 65 (79.3) | 24 (75.0) | 0.621 | 203 (76.9) | 45 (77.6) | 0.910 |  |  |  |
| Age, yrs | 49.8±11.3 | 48.7±8.7 | 0.618 | 47.9±10.1 | 48.5±10.9 | 0.664 |  |  |  |
| BMI | 22.7±2.8 | 22.8±2.8 | 0.933 | 23.0±3.2 | 24.4±4.8 | **0.010** | **1.35** | **[0.33;2.38]** |  |
| Age at onset | 18.9±10.9 | 18.1±10.8 | 0.707 | 17.6±8.0 | 18.7±9.8 | 0.378 |  |  |  |
| MMDs at baseline | 10.4±2.4 | 10.9±2.0 | 0.319 | 20.3±6.4 | 19.4±7.6 | 0.370 |  |  |  |
| MHDs at baseline | 11.1±2.2 | 11.1±2.0 | 0.939 | 23.4±5.4 | 24.4±6.0 | 0.229 |  |  |  |
| Analgesics per month | 13.0±6.9 | 13.9±5.6 | 0.512 | 26.2±21.9 | 32.0±22.5 | 0.068 |  |  |  |
| Medication overuse | - | - | **-** | 228 (86.3) | 52 (89.7) | 0.500 |  |  |  |
| Medication overuse duration, months^#^ | - | - | - | 6 [1-15] | 24 [3-123] | **<0.001 [Z=-3.805]** |  |  |  |
| NRS score at baseline | 7.4±1.4 | 7.3±1.4 | 0.564 | 7.7±1.2 | 7.3±1.3 | **0.021** | **-0.41** | **[-0.77;-0.06]** |  |
| Unilateral pain | 55 (67.9) | 20 (62.5) | 0.584 | 160 (60.6) | 22 (37.9) | **0.003** | **-22.7%** | **[-35.8;-8.60]** |  |
| UAS | 36 (43.9) | 15 (46.9) | 0.774 | 154 (58.3) | 31 (53.4) | 0.496 |  |  |  |
| Allodynia | 44 (53.7) | 17 (53.1) | 0.959 | 162 (61.4) | 33 (56.9) | 0.528 |  |  |  |
| Unilateral pain + UAS | 26 (32.1) | 9 (28.1) | 0.681 | 101 (38.3) | 12 (20.7) | **0.017** | **-17.6%** | **[-28.6;-4.7]** |  |
| Unilateral pain + allodynia | 28 (34.6) | 13 (40.6) | 0.546 | 102 (38.6) | 10 (17.2) | **0.003** | **-21.4%** | **[-31.8;-9.0]** |  |
| Unilateral pain + UAS + allodynia | 16 (19.8) | 8 (25.0) | 0.539 | 81 (30.7) | 5 (8.6) | **<0.001** | **-22.1 %** | **[-30.2;-11.4]** |  |
| Dopaminergic symptoms | 59 (71.9) | 20 (62.5) | 0.326 | 153 (58.0) | 51 (87.9) | **<0.001** | **30.0%** | **[18.3;39.2]** |  |
| HIT-6 score at baseline | 65.6±8.9 | 64.5±5.5 | 0.509 | 67.3±7.2 | 63.3±13.8 | **0.002** | **-3.98** | **[-7.71;-0.23]** |  |
| MIDAS score at baseline^#^ | 26 [12-71.2] | 98 [22-98] | 0.244 | 90 [48-115] | 100 [85-100] | 0.604 |  |  |  |
| Triptan responders | 60 (82.2) | 21 (80.8) | 1.000 | 169 (75.1) | 34 (77.3) | 0.910 |  |  |  |
| Prior treatment failures  *1-2*  *3-4*  *>5* | 4.2±1.7  13 (16.0)  32 (39.5)  36 (44.4) | 4.3±1.7  4 (12.5)  12 (37.5)  16 (50.0) | 0.780  0.831 | 5.0±1.9  17 (6.5)  92 (35.0)  154 (58.5) | 5.3±1.8  1 (1.8)  12 (21.4)  43 (76.8) | 0.187  **0.032 [χ2=6.87]** |  |  |  |
| Pts using concomitant prophylaxis | 36 (43.9) | 21 (65.6) | 0.061 | 142 (53.8) | 33 (56.9) | 0.667 |  |  |  |
| Comorbidities | 1.2±1.2 | 1.0±1.0 | 0.277 | 0.9±1.0 | 1.4±1.2 | **<0.001** | **0.58** | **[0.28;0.88]** |  |
| Pts with ≥1 comorbidity | 54 (65.9) | 20 (62.5) | 0.905 | 143 (54.2) | 44 (75.9) | **0.004** | **21.7%** | **[8.40;33.4]** |  |
| Pts with psychiatric comorbidities | 24 (30.0) | 10 (31.2) | 0.835 | 80 (30.3) | 30 (51.7) | **0.002** | **21.4%** | **[7.40;35.0]** |  |
| Onabotulinum toxin A responders* | 2 (7.7) | 3 (30.0) | 0.197 | 9 (8.0) | 2 (5.3) | 0.833 |  |  |  |
| Erenumab | 70 (85.4) | 30 (93.8) | - | 241 (91.3) | 57 (98.3) | - |  |  |  |
| Galcanezumab | 1 (1.2) | - | - | 3 (1.1) | - | - |  |  |  |
| Fremanezumab | 11 (13.4) | 2 (6.2) | - | 20 (7.6) | 1 (1.7) | - |  |  |  |

Abbreviations: BMI, Body Mass Index; MMD, Monthly Migraine Day; MHD, Monthly Headache Day; UAS, Unilateral cranial autonomic symptoms; NRS, Numerical Rating Scale; HIT-6, Headache Impact Test-6; MIDAS. Migraine Disability Assessment Scale

^#^median [interquartile range, IQR]; ^*^Proportion calculated on the 261 subjects who were treated with onabotulinum toxin A.
